# Shear stress dynamics in root canal irrigation: a systematic review of computational fluid dynamics studies on syringe irrigation with various needle designs

**DOI:** 10.3389/fdmed.2026.1720637

**Published:** 2026-02-09

**Authors:** Niranjan Harikrishna, Nishmitha N. Hegde, Chaithra Lakshmi, Avishikta Banerjee, Kavya Gupta, Mithra N. Hegde

**Affiliations:** 1 Nitte (Deemed to be University), AB Shetty Memorial Institute of Dental Sciences (ABSMIDS), Department of Conservative Dentistry and Endodontics, Mangalore, India; 2 AB Shetty Memorial Institute of Dental Sciences, Nitte (Deemed to be University), Mangalore, Karnataka, India

**Keywords:** computational fluid dynamics, endodontics, fluid dynamics, hydrodynamics, root canal irrigants, root canal preparation, shear strength

## Abstract

**Background:**

The efficacy of endodontic therapy is critically dependent on effective irrigation, which facilitates the removal of debris and biofilm from anatomical regions inaccessible to mechanical instrumentation. Computational Fluid Dynamics (CFD) offers an in silico framework for analyzing irrigant flow dynamics and wall shear stress distribution across varying needle designs and canal morphologies.

**Methods:**

This systematic review was conducted in accordance with PRISMA 2020 guidelines and registered on the Open Science Framework (DOI: 10.17605/OSF.IO/CYNME). A comprehensive search of PubMed, Scopus, and Web of Science through September 2025 identified CFD studies evaluating syringe-based irrigation with diverse needle configurations. Inclusion criteria encompassed in silico investigations assessing WSS within simulated or extracted root canal systems. Data regarding needle type, flow parameters, apical preparation size, and shear stress outcomes were extracted and synthesized qualitatively.

**Results:**

Of the 151 records initially identified, 36 studies met the eligibility criteria, and 10 CFD studies provided needle-specific WSS data. Across these models, open-ended needles consistently produced the highest and most apically concentrated WSS and apical pressure, whereas side-vented needles yielded moderate WSS with comparatively lower pressure. Double side-vented and modified multi-outlet designs demonstrated a reduction in peak stress while enhancing wall coverage. Smaller apical preparations and narrow canal tapers were associated with elevated WSS, while larger preparations attenuated WSS and facilitated smoother flow dynamics. These trends are consolidated within a structured evidence map and a heatmap summarizing the directionality and relative magnitude of WSS across studies.

**Conclusions:**

CFD-based evidence highlights the critical influence of needle geometry and canal morphology on irrigation efficacy and safety. Side-vented needles emerge as the most clinically balanced configuration, while open-ended designs warrant cautious application. Advancing the standardization of CFD protocols and integrating anatomically realistic canal models are imperative for improving translational applicability and informing evidence-based irrigation strategies in contemporary endodontic practice.

**Systematic Review Registration:**

https://doi.org/10.17605/OSF.IO/CYNME.

## Introduction

The enduring efficacy of endodontic therapy fundamentally hinges upon the proficient elimination of microorganisms and associated necrotic debris from the complex root canal architecture, an inherently challenging objective that necessitates meticulous chemo-mechanical preparation and optimized irrigation. Given that anatomical complexities routinely leave 40%–50% of canal walls un-instrumented, the hydrodynamic behavior of the irrigant assumes a pivotal role in achieving effective disinfection and biofilm removal (1, 2). Attaining this clinical objective necessitates a precise understanding of irrigant flow characteristics, including parameters such as velocity, WSS, and apical pressure, which collectively govern the efficacy of canal debridement while concurrently modulating the risk of potentially deleterious periapical extrusion (3, 4).

Computational Fluid Dynamics has emerged as a pivotal in silico methodology, effectively overcoming the limitations inherent in *ex vivo* and *in vivo* assessments by enabling the simulation and detailed analysis of complex, transient fluid dynamics within precisely prepared root canal anatomies, while accounting for a broad spectrum of influential variables, including canal taper, apical preparation dimensions, and irrigant flow rate (1, 5–8).

Within the irrigation protocol, the geometric configurations and functional attributes of the needle, particularly those associated with side-vented, open-ended, and closed-ended designs, constitute a fundamental determinant of the resulting flow dynamics and the generation of WSS necessary for effective biofilm disruption, particularly within anatomically complex and clinically critical regions such as isthmuses and apical ramifications (9–12).

However, despite extensive CFD investigations exploring various parameters (13, 14), persistent gap in knowledge and ongoing debate remain regarding the comparative hydrodynamic efficacy and safety profiles of these distinct needle tip configurations across varying insertion depths and canal morphologies, particularly in relation to the critical balance between maximizing WSS to enhance cleaning efficiency and minimizing apical pressure to prevent irrigant extrusion (15–18).

Consequently, the objective of this systematic review is to rigorously evaluate and synthesize the existing literature on CFD as it relates to the influence of conventional syringe irrigation needle configuration (side-vented, open-ended, and closed-ended) on key hydrodynamic parameters, with particular emphasis on wall shear stress. The review seeks to elucidate evidence-based performance differentials, resolve persistent clinical ambiguities, and provide informed guidance for the refinement of endodontic irrigation protocols.

## Methods

The systematic review was done according to the PRISMA 2020 guidelines which are followed for systematic reviews as given by Page et al. (19).

### Registration

The protocol was registered in the Open Science Framework (OSF) Database, with the registration DOI: https://doi.org/10.17605/OSF.IO/CYNME.

### Data sources and search strategy

A comprehensive and systematic search was executed across the following electronic databases: PubMed, Scopus, and Web of Science. The search process was conducted up to the 29th of September 2025, without imposing any restrictions regarding the date, language of publication was restricted only to English. The search queries were formulated utilizing a synthesis of primary keywords and MeSH terms pertinent to root canal irrigation, conventional needle irrigation, computational fluid dynamics, and shear stress analysis. Full database-specific search strategies, including all Boolean operators, field tags, and any filters, are provided in the Supplementary Data S1 in accordance with PRISMA-S guidelines.

### Study selection and outcome measurement

**Figure 1:**
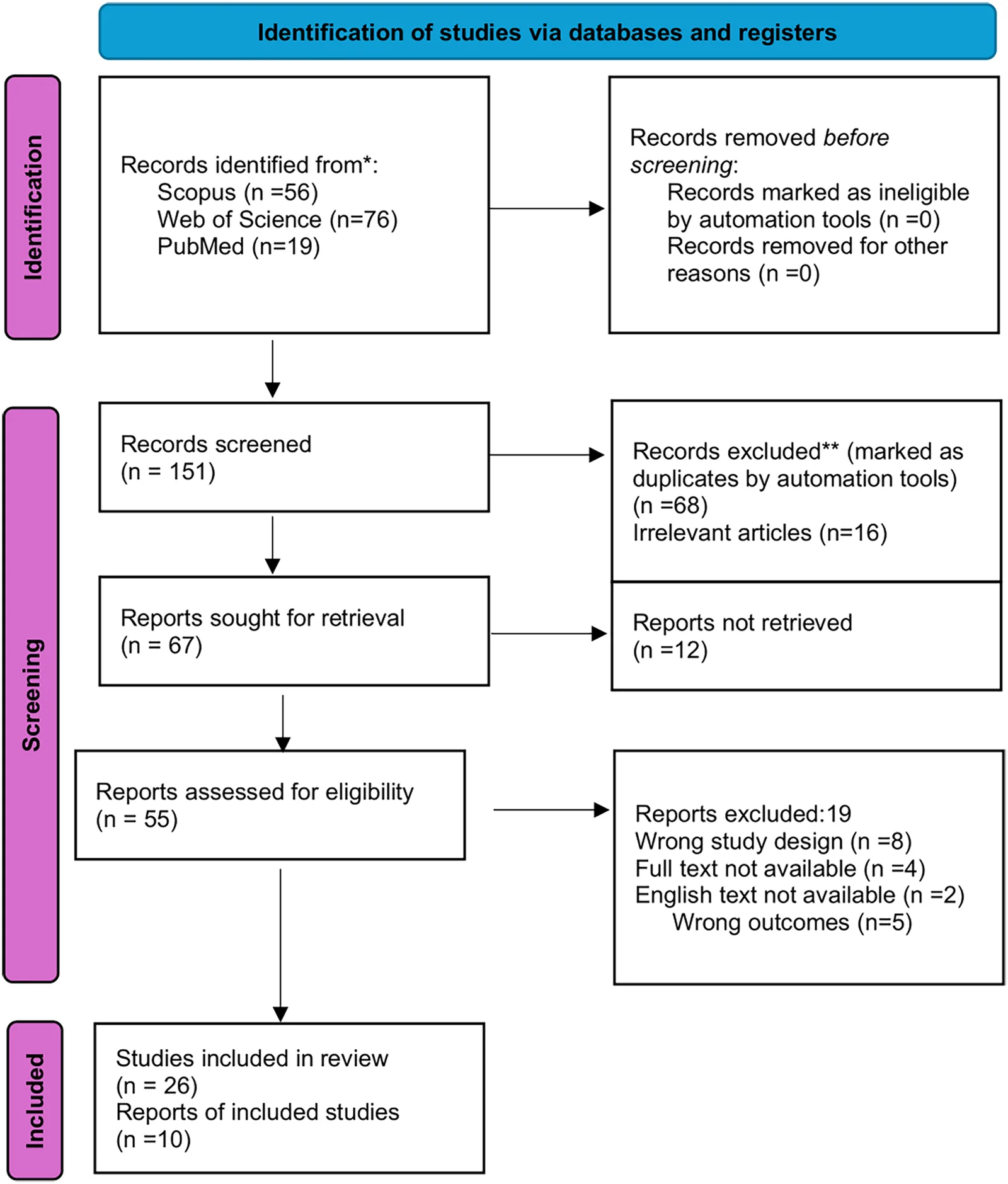
PRISMA 2020 flow diagram for new systematic reviews which included searches of databases and registers only.

Two researchers (NHK and KG) conducted an independent evaluation of the titles and abstracts utilizing a web-based systematic review tool, Covidence. Any inconsistencies that arose were reconciled through a consensus process involving a third researcher (MNH). The conclusive study methodology was illustrated through a PRISMA flow diagram (19) Figure 1.

Inclusion criteria: A PEOS format was used to formulate the inclusion criteria as follows,

- Participants (P): Root canal system (simulated and extracted)
- Exposure (E): Conventional syringe irrigation with various needle designs
- Outcome (O): Shear stress applied onto the root canal walls
- Study design (S): computational studies or in silico studies

Exclusion criteria: The following exclusion criteria were used.

Other types of irrigation methods (negative pressure, ultrasonic irrigation, sonic irrigation), results not including shear stress as one of the parameters, *in-vitro* studies without CFD validations, studies lacking CFD methodology, reviews, editorials, or conference abstracts without full data, studies without needle-specific modelling or shear stress outcomes ⁠and non-English publications.

### Outcome measure

The primary outcome measure of this study was to evaluate the shear stress applied onto the canal walls by various needle designs during conventional syringe irrigation.

The secondary outcome measures of this study were to evaluate the effect of apical preparation size and canal taper on shear stress applied onto the canal walls.

### Data extraction

The following parameters were included: Objectives of the study, methods used, findings, practical implications, limitations, results and conclusions Supplementary data S2, S3.

### Assessment of risk of bias

There is no validated risk of bias tool for CFD irrigation studies in Endodontics. Hence we adapted the STRESS-CFD (Simulation in Research: Standards for reporting CFD studies) reporting framework to construct a structured methodological quality assessment specific to root canal CFD simulations. The Final tool comprised 7 domains reflecting key stress CFD items:

1. Geometry definition and anatomical realism
2. MeSH generation and numerical resolution
3. Boundary and initial conditions
4. Fluid properties and flow specifications
5. Outcome reporting (wall shear stress, apical pressure, and related parameters)
6. Numerical solution strategy and transparency (solver, turbulence model, convergence)
7. Validation (mesh independence, comparison with experimental or previously validate models)

**Figure 2:**
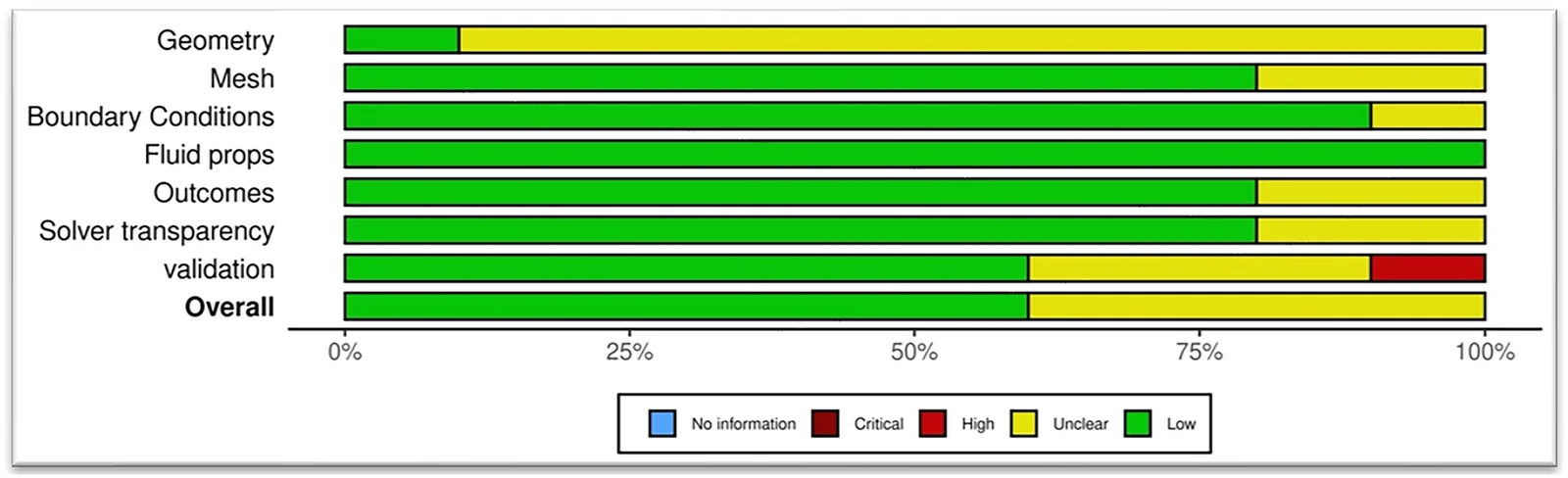
Risk of bias across domains.

**Figure 3:**
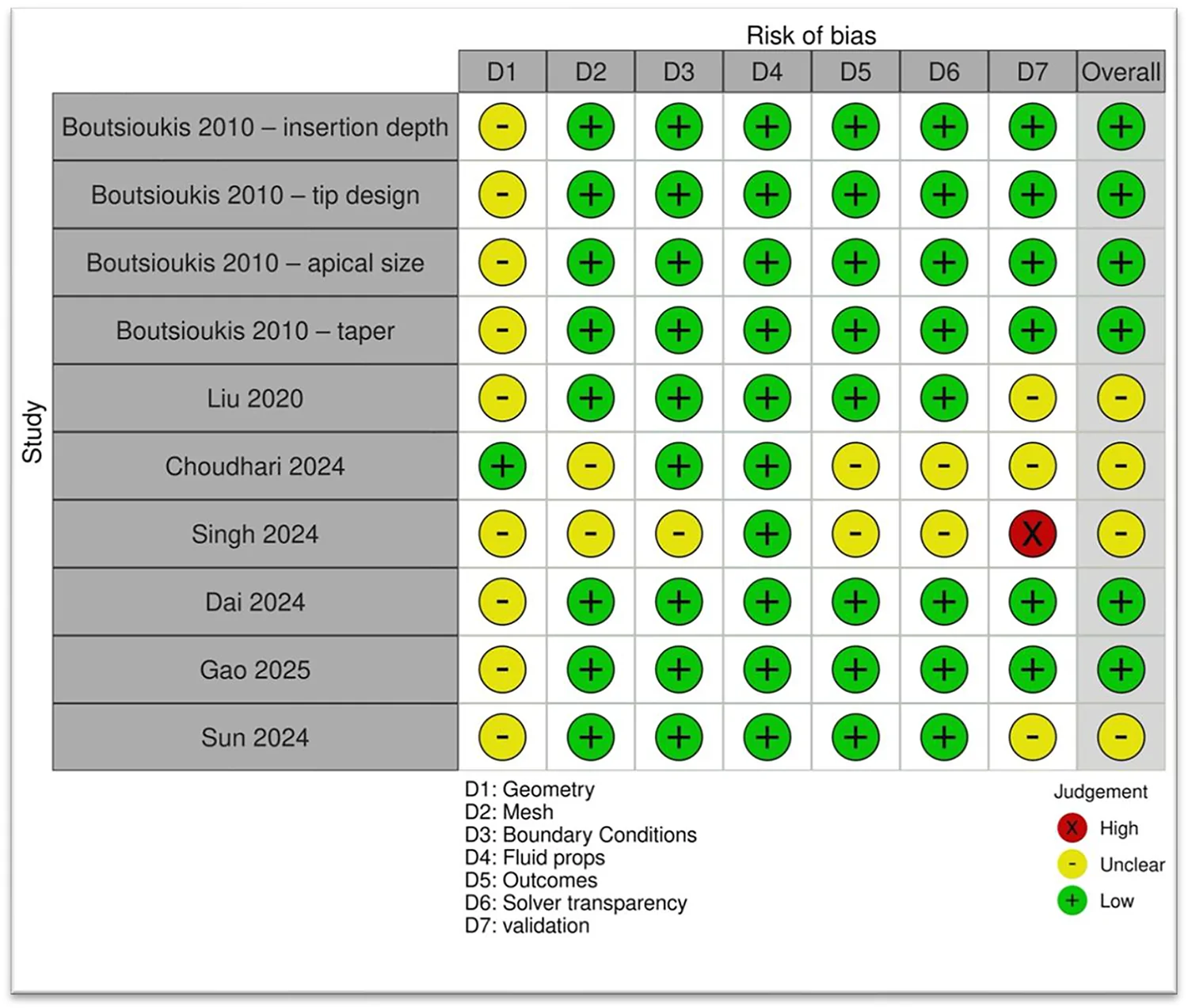
Risk of bias across included studies.

Any discrepancies that arose were communicated through dialogue and consensus or by seeking the input of a third reviewer (MNH). The outcomes of the quality assessment were classified into three categories: “Low”, “Moderate”, and “High” risk of bias. Supplementary data S4, Figures 2, 3.

### Structured evidence synthesis

**Figure 4:**
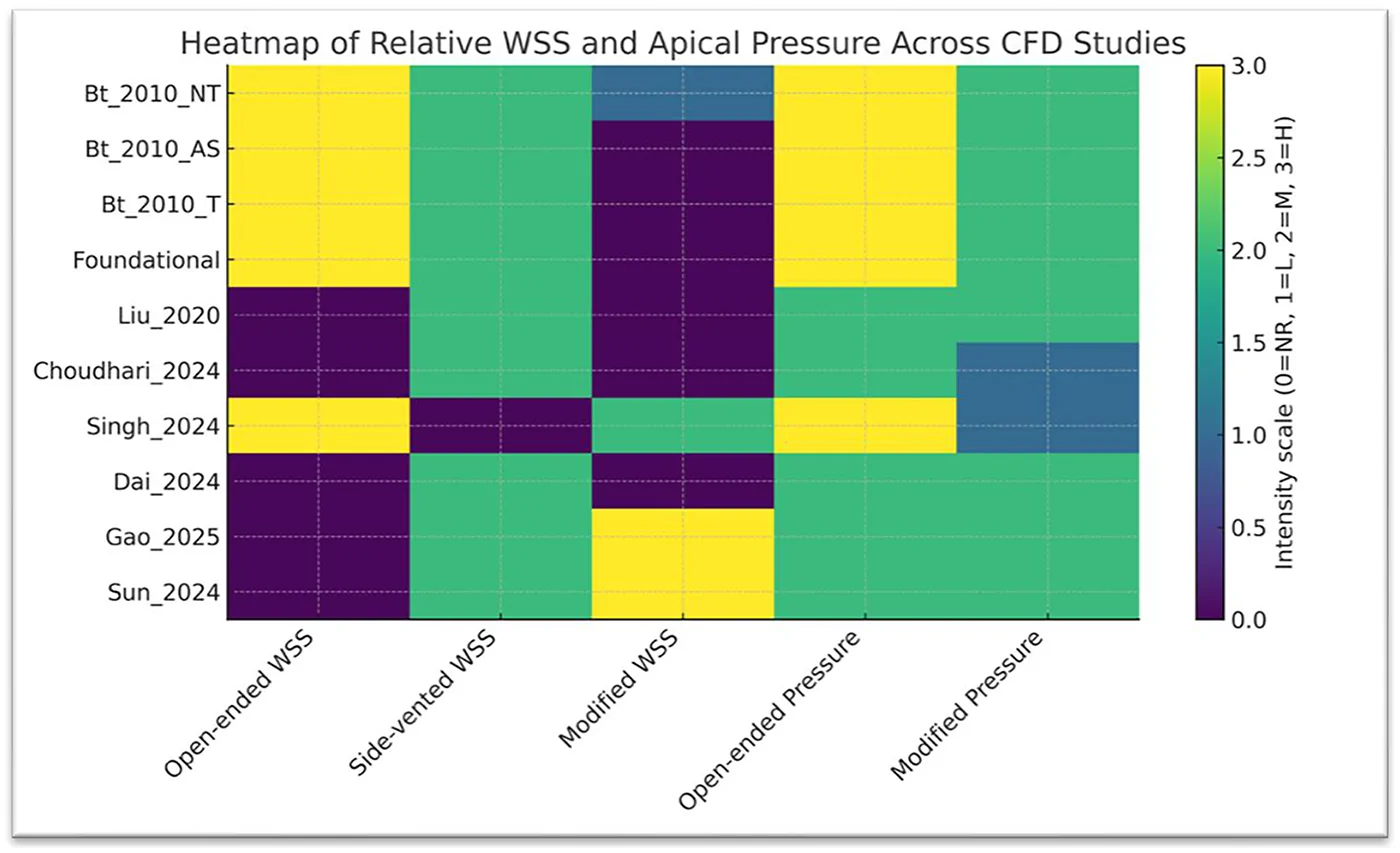
Heatmap of relative wall shear stress (WSS) and apical pressure across ten computational fluid dynamics (CFD) studies of syringe-based irrigation.

Owing to substantial heterogeneity in canal geometries, boundary conditions, and outcome definitions, a formal meta-analysis was not feasible. Instead, we performed a structured evidence synthesis using a vote-counting approach. For each key parameter (needle design, apical preparation size, taper, and tip-to–working length offset), we coded the direction of effect on wall shear stress (increase, decrease, or no clear change) across all studies that modelled that comparison. These data were summarized in an evidence map and in a heatmap-style matrix to illustrate the relative magnitude and consistency of WSS and apical pressure across needle designs Supplementary data S5 and Figure 4.

The heatmap summarizes the direction and relative magnitude of WSS and apical pressure reported for different needle designs (open-ended, side-vented, and modified/multi-vented designs) across all studies included in the systematic review. Each cell represents an intensity score derived using a structured vote-counting approach (0 = not reported; 1 = low; 2 = moderate; 3 = high).

Columns represent performance domains (Open-ended WSS, Side-vented WSS, Modified-needle WSS, Open-ended Pressure, Modified-needle Pressure). Rows correspond to the ten included CFD studies: Boutsioukis et al. 2010 (needle type, apical size, taper), Boutsioukis foundational CFD model, Liu et al. 2020, Choudhari et al. 2024, Singh et al. 2024, Dai et al. 2024, Gao et al. 2025, and Sun et al. 2024 (13, 16, 20–22).

The heatmap highlights consistent trends across heterogeneous CFD models,

- Open-ended needles generated the highest peak WSS and apical pressure
- Side-vented needles produced moderate WSS with reduced apical pressure
- Modified multi-vented designs (double side-vented, sealed-bevel, composite outlets)

achieved lower peak WSS while improving wall coverage at clinically safer pressure levels. This structured visual synthesis supports interpretation of the aggregated CFD literature and complements the evidence map presented in Supplementary data S5.

## Results

### Search results

The electronic search across PubMed (*n* = 19), Scopus (*n* = 56), and Web of Science (*n* = 76) yielded a total of 151 records. These citations were imported into Covidence, where 65 duplicates were removed by automation tools and 19 records were excluded as clearly irrelevant, leaving 67 records for title-and-abstract screening. A total of 55 full-text articles were subsequently assessed for eligibility, as documented in the PRISMA-2020 flow diagram. Following full-text evaluation, 19 reports were excluded for the following reasons consistent with PRISMA-defined exclusion categories:

1. wrong study design (*n* = 8);
2. full text not available (*n* = 4);
3. article not available in English (*n* = 2); and
4. wrong outcomes, including absence of shear-related parameters (*n* = 5).

Ultimately, 36 studies fulfilled the eligibility criteria and were included in the qualitative synthesis. 10 CFD studies provided extractable, needle-specific wall shear stress data within root canals and were included in the primary outcome analysis. The remaining 26 included studies contributed contextual information on apical pressure, vapor-lock behaviour, canal geometry, numerical modelling assumptions, and model-validation strategies, forming part of the narrative synthesis. The complete selection process is illustrated in the PRISMA-2020 flow diagram.

Most CFD studies showed low risk of bias in mesh specification, boundary conditions, and solver transparency, but several had some concerns regarding anatomical realism of the canal geometry and limited experimental validation, particularly for newer optimization studies.

Domain-level scores for all 10 included CFD studies are presented in Supplementary Data S1.

Overall methodological quality was high, with most studies achieving ≥5/7 on the STRESS-CFD–adapted scoring system. Lower scores were typically attributable to incomplete reporting of validation procedures or qualitative-only WSS outputs.

### Study characteristics

#### Populations

This study is in silico based and we considered our population to be simulated root canals either digitally or scanned.

#### Exposure

Exposure of canal models to various needle designs (close end, open end, side vented etc) for syringe irrigation.

#### Outcome assessment

##### Primary objective: needle design effects on shear stress

The primary objective of this systematic review was to assess the influence of needle design on the generation and distribution of WSS during syringe-based root canal irrigation, as simulated by validated CFD models. Among the configurations studied, open-ended needles, including flat, beveled, and notched variants consistently produced the highest WSS values, with a range spanning from 480 Pa to a maximum of 129,000 Pa. The peak value was reported by Singh et al. (2024), highlighting the capacity of open-ended needles to deliver intense fluid jets directly toward the apex, thereby increasing local velocity and shear force. While this design offers the highest debris removal potential, it also demands careful apical pressure management to prevent extrusion (16)..

Side-vented needles, particularly those with a single lateral outlet, demonstrated moderate yet consistent WSS generation, ranging from 660 Pa to 11,000 Pa. Studies by Boutsioukis et al. (2010) provided robust evidence for the efficacy and safety profile of this configuration (23). The lateral jet mechanism facilitates controlled wall impact and reduced apical pressure, offering a balanced cleaning strategy that has gained wide clinical acceptance. In contrast, double side-vented needles (DSVN) exhibited lower peak WSS values than their single-outlet counterparts. Singh et al. (2024) noted a significant reduction in wall stress with DSVN designs, attributing this to distributed flow patterns that diminish localized shear concentration. This design is particularly suitable for cases involving delicate apical tissues or widened foramina (16).

Innovative and modified needle designs, including those evaluated by Dai et al. (2024) and Sun et al. (2024), introduced novel aperture geometries and composite structures aimed at optimizing flow control (13, 22). These designs consistently achieved WSS values exceeding the proposed clinical threshold of 100 Pa, supporting their potential for effective cleaning. By incorporating multi-outlet flow paths and structural adaptations, such needles aim to deliver tailored stress distributions while enhancing wall coverage and maintaining apical safety margins.

##### Secondary objective: apical preparation size effects on shear stress

The secondary objective was to evaluate the effect of apical preparation size and canal taper on shear stress outcomes. Narrow preparations, particularly those at ISO size 25, were associated with elevated WSS reaching up to 11,000 Pa. Boutsioukis et al. (2010) attributed this to increased fluid velocity and impact force within confined geometries. Conversely, larger apical sizes (ISO 35–55) demonstrated a progressive decline in WSS, with values dropping from approximately 1,000 Pa to as low as 200 Pa. This inverse relationship was corroborated by Boutsioukis et al. (2010), who reported that enlarged canal spaces facilitate smoother flow transitions and energy dissipation. Canal taper also played a critical role, canals with minimal taper (2%) exhibited the highest WSS due to tighter luminal constraints, while wider tapers (6%–8%) produced more uniform but less intense shear profiles (23).

According to Sujith et al, the evidence suggests that an apical preparation of ISO 30 with a taper of 4%–6% may offer the most clinically advantageous balance between efficient irrigant delivery and the minimization of apical extrusion risk. This preparation permits adequate fluid penetration and stress generation while preserving structural safety. These insights reinforce the necessity of tailoring both needle selection and canal preparation to the anatomical and procedural demands of each case (24).

This review was restricted to syringe-based positive-pressure irrigation systems according to predefined eligibility criteria. Modalities such as ultrasonic activation, sonic irrigation, and negative-pressure systems were excluded at the protocol stage. Therefore, any references to these systems in the discussion serve solely as contextual background and do not reflect comparative evaluation or evidence synthesis. While CFD offers valuable mechanistic insights, clinical outcomes such as disinfection efficacy and healing cannot yet be reliably inferred from WSS values alone. Future work integrating CFD with experimental or patient-level data is essential (1, 5).

## Discussion

### Clinical applications

Recent technological advancements in CFD, combined with ex-vivo and *in-vitro* study data, have underscored the critical role that irrigation needle design plays in the success of endodontic treatments. The needle choice affects both the efficiency of canal cleaning and the procedural safety, making it a key consideration in a clinical setting. Other irrigation modalities, including *in-vitro* or ex-vivo systems, were not part of the included evidence and are mentioned only to contextualize CFD findings rather than to inform comparative conclusions.

#### Side-vented needles

Side-vented needles are widely favoured due to their optimal balance between cleaning performance and patient safety. Stronger shear forces are generated along the canal walls by these needles which effectively dislodges dentinal debris while maintaining low apical pressure. This reduces the risk of extrusion of the irrigant solution into the surrounding periapical tissues (12, 25, 26).

They are particularly useful in challenging cases such as immature teeth or blunderbuss canals. usually, the clinician positions these needles 1–3 mm short of the apex to maximize cleaning and to avoid apical extrusion (3, 14).

Additionally, side-vented designs enhance lateral irrigant flow, making them effective in complex canal geometries like curved canals and isthmuses (2, 11).

#### Closed-ended and negative pressure systems

Closed-ended needles and systems like EndoVac are engineered to maintain low pressure at the tip, significantly reducing the risk of irrigant extrusion beyond the root canal (15, 27). These systems are usually recommended for anatomically complex canals or when patient safety is of utmost importance.

Novel designs, such as double side-vented needles with surface modifications like protrusion or dimples, have shown improvement in irrigant distribution and cleaning efficiency without affecting safety. These are particularly beneficial in cases involving persistent biofilms or intricate canal structures (9, 22, 28) Negative-pressure irrigation systems were excluded according to the predefined protocol; therefore, the above statements are provided only as contextual background and do not represent comparative analysis within this review.

#### CFD-guided clinical protocols

CFD research has improvised clinical protocols by offering potential insights into best needle insertion depth, irrigant solution flow rates, and canal shaping strategies. Advanced imaging technologies like micro-CT have confirmed these protocols, enhancing their precision and reliability. To maintain protocol fidelity, interpretations in this review are restricted to syringe-based CFD models; references to external modalities serve only as scientific context.

#### Clinical implications for root canal irrigation

1. Maximizing Debris Removal: Open-ended needles used in adequately prepared canals (ISO size ≥30) deliver high shear stress, which enhances cleaning efficiency while supporting safety margins (1).
2. Ensuring Safety in Sensitive Cases: Side-vented needles are ideal for minimally prepared canals, offering effective cleaning with reduced risk of excessive wall stress or irrigant extrusion (16).
3. Achieving Optimal Balance: Emerging needle designs with flow control features stand for the future of irrigation. These designs aim to deliver targeted shear stress where needed while protecting delicate canal walls (22).
4. Preparation Strategy for Effective Irrigation: To help efficient irrigation, canals should be enlarged to at least ISO size 30 with a taper of 4%–6%. This preparation allows for effective irrigant flow and manageable shear stress levels, refining both cleaning and safety (24).

It is important to note that the recommendation for canal enlargement to ISO size 30 with a 4%–6% taper is not derived from a formal meta-analytic synthesis. Rather, it reflects a synthesis of observed flow behavior trends across studies, coupled with expert interpretation based on hydrodynamic principles. While moderate enlargement appears to facilitate favorable WSS distribution and minimize apical extrusion risk, the evidence remains indirect and should be interpreted within the broader context of anatomical variability and clinical judgment.

#### Mechanism of action: role of shear stress

Higher shear stress improves irrigation outcomes by:

- Mechanically removing biofilm and debris from canal walls
- Enhancing irrigant penetration into dentinal tubules
- Promoting fluid mixing and replacement
- Activating chemical irrigants through turbulent flow

#### Clinical recommendations

Needle selection should be based on canal anatomy:

- Open-ended needles are suitable for robust canals requiring aggressive cleaning.
- Side-vented needles are preferred for conservative approaches and delicate anatomies.
- Irrespective of the design, adequate apical preparation is necessary to balance cleaning efficiency with patient safety.

However, these clinical insights must be interpreted within the modeling constraints of the included studies. The absence of anatomical realism in most CFD models limits the certainty with which these wall shear stress patterns can be extrapolated to patient scenarios. Until greater anatomical fidelity is routinely incorporated into simulation frameworks, WSS thresholds and flow patterns derived from simplified models should be considered mechanistic indicators rather than prescriptive clinical targets.

### Strengths

The existing corpus of inquiry examining the impact of various needle configurations on root canal irrigation reveals several significant strengths, particularly in the use of computational fluid dynamics alongside experimental techniques. A central advantage is the compelling demonstration of how needle tip geometry affects wall shear stress distribution and flow dynamics of the irrigant. Numerous investigations consistently indicate that side-vented needles yield localized high wall shear stress while maintaining lower apical pressure, thereby promoting safety and facilitating effective irrigant exchange (12, 25, 27).

Conversely, open-ended needles tend to generate elevated irrigant replacement and shear stress levels, potentially enhancing cleaning efficiency, particularly in the apical region (2, 16).

The scholarly literature further underscores the potential of innovative designs, such as double side-vented needles featuring dimples or protrusions, in optimizing flow dynamics and shear stress while minimizing the risk of extrusion (9, 22).

Another significant advantage lies in the integration of advanced CFD modelling with empirical validation. Numerous investigations utilize micro-CT-based realistic canal modelling, corroborating CFD predictions through micro-particle image velocimetry (micro-PIV) or *ex vivo* datasets, thereby enhancing the reliability and clinical relevance of their findings (18).

The inclusion of both transient and steady-state simulations enables the exploration of a wide range of clinical scenarios, considering the effects of needle design, flow rates, insertion depth, and canal morphology on irrigation behaviour (15, 27).

The literature further illustrates robustness in addressing clinically pertinent variables such as needle gauge, flow rate, canal taper, and preparation size, establishing connections to irrigation effectiveness and safety (18, 23, 24).

The research reveals data about the importance of needle insertion depth and root canal anatomy in influencing root canal irrigation dynamics. Investigations consistently emphasize that optimal needle placement, generally 1–3 mm short of the working length, strikes a balance between maximizing wall shear stress and irrigant renewal while mitigating apical pressure and extrusion risk (12, 14, 25).

Lastly, the thematic diversity within the literature, including the effects of needle tip geometry, safety profiles associated with apical pressure and extrusion risk, as well as the impact of canal curvature and anatomy, offers a holistic understanding of the multifactorial nature of root canal irrigation. This diversity is further bolstered by the investigation of innovative needle designs and passive flow control mechanisms, which represent promising pathways for future enhancement (22, 28).

## Limitations

While computational fluid dynamics and experimental studies have significantly advanced our understanding of the effects of various needle designs on root canal irrigation, many limitations exist that constrain the generalizability and clinical translation of current findings.

A predominant limitation is the reliance on simplified or idealized root canal geometries in many CFD models, such as straight conical canals, which do not fully capture the anatomical complexity and surface irregularities of real root canals (14). While this issue has been acknowledged, the majority of included CFD studies (e.g., Boutsioukis et al., 2010; Singh et al., 2024; Dai et al., 2024) employed simplified conical or cylindrical canals that do not account for anatomical irregularities, lateral canals, or surface roughness (1, 13, 16). This introduces a significant translational gap between in silico flow dynamics and clinical irrigation outcomes. Only few studies, such as Gao et al. (2025) and Sun et al. (2024), utilized micro-CT-derived anatomies or validation against experimental datasets (22, 29). As such, generalization of WSS values and apical pressure behavior must be approached with caution, particularly in narrow, curved, or bifurcating canals that are not represented in most models.

This simplification may underestimate flow heterogeneity, wall shear stress distribution, and the challenges posed by anatomical variations, including isthmuses, apical ramifications, and canal curvature (11, 12).

Another significant constraint is the limited experimental validation of CFD simulations**.** Several studies lack comprehensive *in vitro* or *ex vivo* confirmation of predicted flow patterns, shear stress, and apical pressure values, raising concerns about the accuracy and reliability of computational results (9, 30).

This methodological gap affects confidence in the clinical applicability of CFD-based recommendations.

The narrow range of needle designs investigated in most research further restricts the scope of conclusions. Many studies focus primarily on conventional side-vented, open-ended, and closed-ended needles, often excluding newer or innovative designs with complex flow control features such as dimples, protrusions, or multiple lateral apertures (16, 22, 28).

As a result, the full spectrum of irrigation performance and safety profiles stays insufficiently characterized.

Additionally, there is no universally accepted or experimentally validated apical pressure threshold for irrigant extrusion, complicating safety assessments and the ability to definitively evaluate extrusion risk and needle safety across studies (31, 32).

This inconsistency undermines the development of standardized clinical guidelines.

The limited consideration of root canal curvature in many studies further reduces the applicability of findings to clinical scenarios, as severe or complex canal curvatures can significantly alter flow patterns and shear stress (14, 33).

Similarly, most CFD analyses do not incorporate multiphase flow dynamics or detailed interactions between irrigants and biofilms, limiting insights into actual cleaning efficacy and biofilm removal mechanisms (3, 34, 35).

Moreover, the use of small sample sizes and artificial canals in experimental studies limits generalizability due to anatomical variability among patients (2, 7, 14, 25, 27).

Finally, several investigations focus on fixed flow rates and irrigant properties, neglecting the impact of varying flow rates and different irrigant viscosities or chemical properties on irrigation dynamics, which constrains applicability to diverse clinical settings (5).

The effect of needle tilting angle and lateral positioning within the canal is also underexplored, despite its potential influence on flow distribution and shear stress (10)..

In summary, while CFD and experimental studies have offered valuable insights into needle design effects on root canal irrigation, future research must address these limitations by incorporating realistic canal anatomies, comprehensive experimental validation, broader needle design exploration, and multifactorial clinical conditions to enhance the reliability and clinical relevance of findings.

### Future directions

Despite significant advancements in CFD modelling and experimental validation, several critical gaps and opportunities are still in optimizing root canal irrigation needle design and translating these findings into clinical practice.

### Standardization of needle design and testing protocols

Current research shows considerable variability in needle geometries, aperture configurations, and passive flow control features across studies, which limits direct comparability and clinical translation. Future research should prioritize the development of standardized design parameters and systematic testing protocols for side-vented, open-ended, and closed-ended needles. Such standardization will facilitate meta-analyses, regulatory approval, and the clinical adoption of innovative needle designs (13, 22, 28).

### Integration of realistic root canal anatomy in CFD models

Many CFD studies still rely on simplified or idealized canal geometries, neglecting complex anatomical features such as isthmuses, apical ramifications, and canal wall irregularities. Incorporating high-resolution micro-CT-based 3D reconstructions of diverse root canal anatomies into CFD simulations will yield more clinically relevant insights into irrigant flow, wall shear stress, and vapor lock formation, ultimately improving irrigation efficacy and safety (11–13).

### Advanced modelling of vapor lock and multiphase flow

Vapor lock is still a significant barrier to effective irrigant penetration, yet current CFD models inadequately simulate multiphase flow and vapor lock dynamics. Future studies should develop multiphase CFD models that incorporate air-liquid interfaces and validate these with experimental vapor lock visualization, thereby informing needle design and irrigation protocols that better address this clinical challenge (3, 12).

### Optimization of needle insertion depth in curved and complex canals

The interaction between needle insertion depth, canal curvature, and needle flexibility is underexplored. Future research should investigate these combined effects using CFD models with flexible needle geometries and real canal curvatures, assessing their impact on wall shear stress, irrigant replacement, and extrusion risk. This will be particularly valuable for optimizing irrigation in anatomically challenging cases (14, 36).

### Incorporation of turbulence, transient flow, and operator technique

Most CFD studies assume steady, laminar flow, neglecting the effects of turbulence, transient irrigation, and needle tilting angles, all of which can significantly influence irrigant mixing, wall shear stress, and apical pressure. Incorporating transient and turbulence modelling, as well as simulating dynamic operator manoeuvrers, will provide a more comprehensive understanding of irrigation performance and safety (5, 10).

### Establishment of clinically relevant apical pressure thresholds

There is currently no universally accepted apical pressure threshold for safe irrigation, complicating safety assessments, and clinical recommendations.

Future research should aim to establish clinically validated apical pressure thresholds through combined CFD, *ex vivo*, and *in vivo* studies, considering periapical tissue compliance and patient-specific factors (17, 31).

### Evaluation of innovative needle designs for biofilm removal

Emerging needle designs incorporating multiple side vents, dimples, or protrusions show promise in enhancing irrigant distribution and wall shear stress without increasing extrusion risk. CFD and *in vitro* biofilm removal studies are needed to compare these novel geometries under clinically relevant flow rates and anatomies, with a focus on their efficacy in disrupting biofilms in complex canal regions (9, 22, 28).

### Multiphysics modelling and clinical translation

Future models should combine fluid dynamics with chemical kinetics of irrigants, biofilm mechanical properties, and tissue interactions to better predict clinical irrigation outcomes. Additionally, collaborative efforts between researchers, clinicians, and manufacturers are essential to translate virtual needle designs into clinically practical products, ensuring thorough experimental and clinical validation.

The addition of a vote-counting evidence map and heatmap-style synthesis helped us identify consistent trends across heterogeneous CFD models, particularly the trade-off between high WSS and apical pressure in open-ended designs vs. safer multi-outlet needles.

## Conclusion

This systematic review synthesized CFD evidence to evaluate how needle design, apical preparation size, and canal taper influence WSS during syringe-based root canal irrigation. The primary findings indicate that open-ended needles consistently produce the highest WSS which is beneficial for enhanced debris removal in well-prepared canals (ISO size ≥30), but pose an elevated risk of apical extrusion. In contrast, side-vented needles demonstrate moderate but clinically sufficient WSS with reduced pressure, making them better suited for anatomically complex or minimally prepared canals. These CFD-derived insights offer mechanistic clarity into needle performance under idealized conditions.

Clinically, the recommendation to prepare canals to ISO 30 with a 4%–6% taper, although not drawn from meta-analytic data it stems from hydrodynamic patterns observed in the reviewed simulations and should be interpreted as expert-driven guidance rather than a definitive protocol. The secondary objective of assessing the role of canal morphology revealed that narrower preparations amplify WSS, while broader tapers promote uniform stress distribution at the cost of peak efficiency.

While these findings support the individualized selection of irrigation protocols, they should be contextualized within the limitations of in silico models. Translating CFD data into clinical decisions requires caution, as most models lack anatomical fidelity and experimental validation. Upcoming research should focus on integrating anatomically realistic geometries into CFD models, correlating CFD metrics with experimental and clinical outcomes, and developing standardized validation protocols. Such advancements will refine irrigation strategies and bridge the gap between simulation and patient care.

## Data Availability

The original contributions presented in the study are included in the article/Supplementary Material, further inquiries can be directed to the corresponding author.
